# Supplementary material for: Alkanes increase the stability of early life membrane models under extreme pressure and temperature conditions
Source: Commun Chem. 2021 Feb 26;4:24. doi: 10.1038/s42004-021-00467-5 (PMC9814696; doi:10.1038/s42004-021-00467-5)
Supplement: Supplementary file 1 — Supplementary Information [file 42004_2021_467_MOESM1_ESM.pdf]

Alkanes increase the stability of early life membrane models under extreme pressure and temperature conditions

Loreto Misuraca[1,2], Bruno Demé[2], Philippe Oger[3], Judith Peters[1,2]*

[1] Univ. Grenoble Alpes, CNRS, LIPhy, 38000 Grenoble, France
[2] Institut Laue - Langevin, F-38042 Grenoble Cedex 9, France
[3] Univ Lyon, INSA Lyon, CNRS UMR5240, Villeurbanne, France
*corresponding author: jpeters@ill.fr

Supplementary Information

Supplementary Note 1: SAXS measurements at T = 5 °C

Supplementary Figures 1, 2, 3 show the SAXS curves collected at T = 5 °C in the pressure range 1 ≤ p ≤ 1000 bar. A number of features are present and tell about the effect of pressure on the investigated system at low temperature. The C10 mix sample (Supplementary Figure 1 (a)) shows the presence of a very small correlation at q ≃ 0.08 Å^{-1} (d-spacing ≈ 78.5 Å). The presence of a second order at q ≃ 0.16 Å^{-1} = 2 × 0.08 Å^{-1} proves that this is a lamellar phase. By comparing the curves at p = 1 bar and T = 5 – 20 °C (Supplementary Figure 1 (b)), one can see that a coexisting swollen phase 0.05 – 0.06 Å^{-1} (d-spacing ≈ 105 – 125 Å) is probably present also at the lowest temperature, although it is too weak to be resolved.

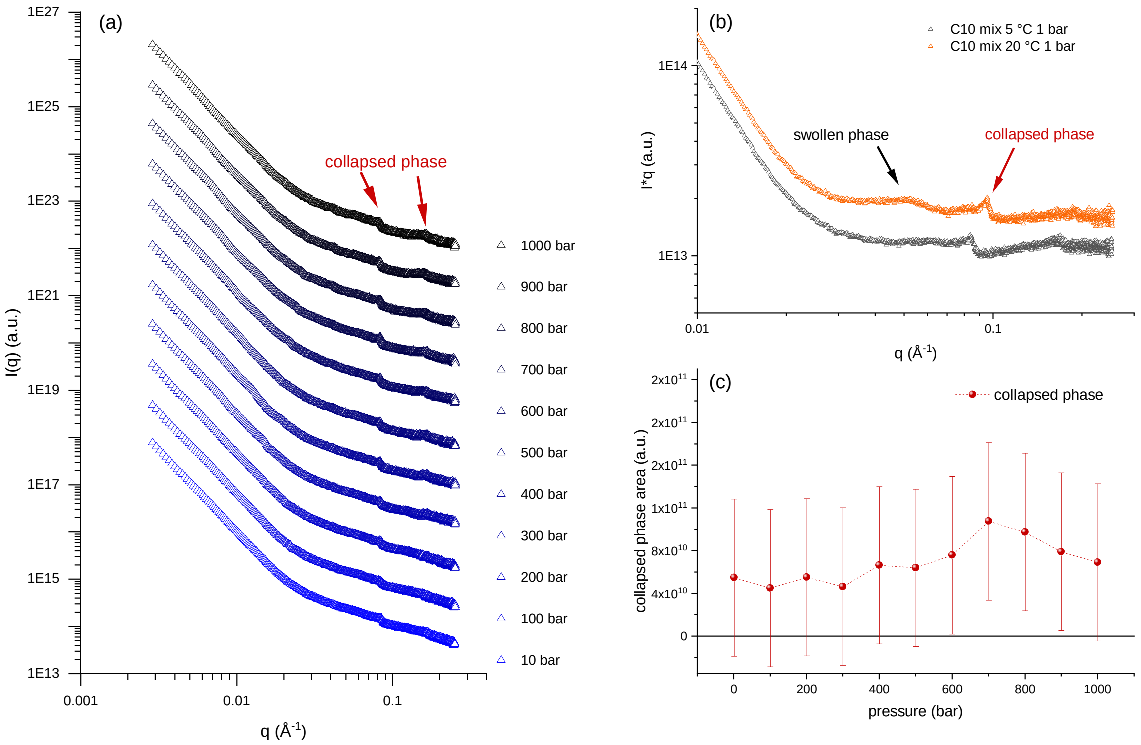

Supplementary Figure 1. C10 mix at 5 °C. a) SAXS data of sample C10 mix, T = 5 °C, at all measured pressure points. b) Comparison of C10 mix curves at T = 5 and 20 °C respectively, in a I*q representation that flattens the curve decay in the surrounding of the membrane correlations. c) Trend with pressure of the peak integrated intensity corresponding to the collapsed phase.

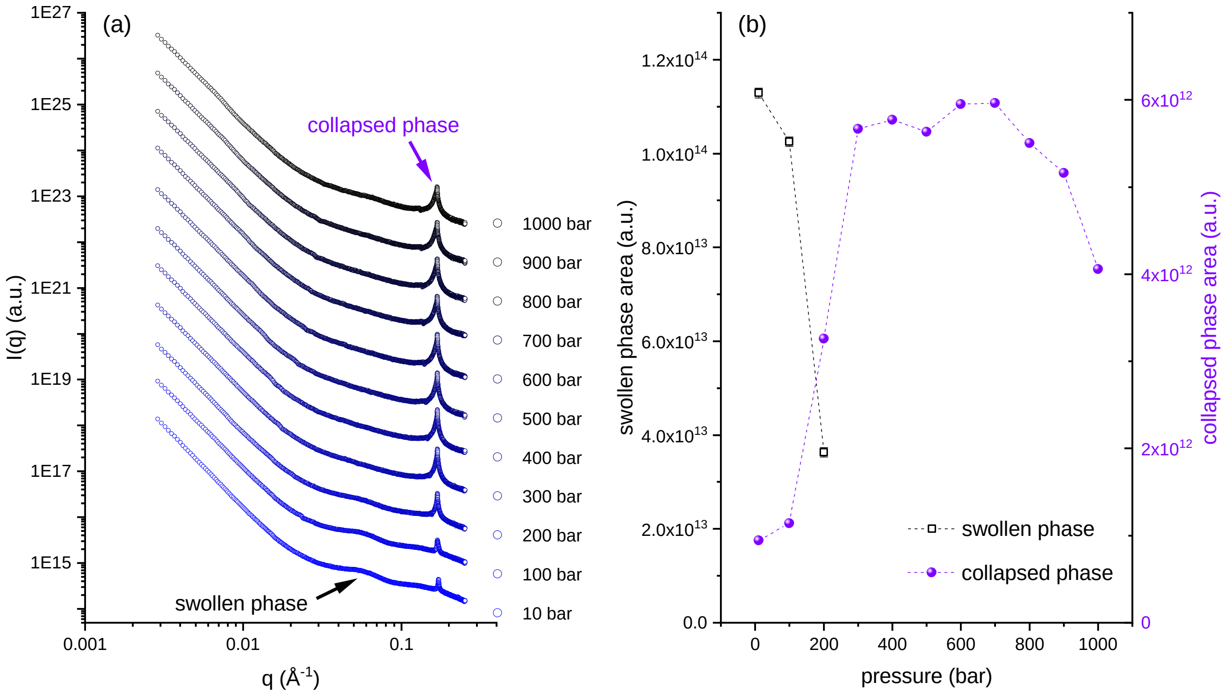

Supplementary Figure 2. C10 mix + 2% eicosane at 5 °C. a) SAXS data of sample C10 mix + 2% eicosane, T = 5 °C, at all measured pressure points. b) Trend with pressure of the peak integrated intensity corresponding to the swollen and the collapsed phase respectively.

The intensity of the peak at q ≃ 0.08 seems to be kept constant at varying pressure, although the very low intensity leaves a high relative uncertainty to its value (Supplementary Figure 1 (c)).

The situation is significantly different for the two other samples containing the alkanes, namely the eicosane and the squalane (Supplementary Figures 2 (a) and 3 (a)). Here, the T = 5 °C curves at p = 10 bar show a well resolved coexistence between the broad swollen phases (centred at q ≃ 0.056 - 0.088 $Å^{-1}$ → d-spacing ≃ 112 – 71 Å, respectively) and well-defined sharp peaks (q ≃ 0.17 $Å^{-1}$ → d-spacing ≃ 37 Å in both cases) which indicates collapsed phases. Upon pressure increasing, both samples show a decrease of the swollen phase signal, accompanied by an increase of the collapsed phase intensity (Supplementary Figures 2 (b) and 3 (b)). The observed phenomenon implies, from one hand, that the collapsed phase detected on the C10 mix sample is likely of a different nature than the one found on the alkane-including samples (a deduction that is also validated by comparing the d-spacings). The fact that the peak of the collapsed phase is observed at a constant q-value allows us to link qualitatively the intensity of the correlation with the volume fraction of sample in that phase. Since the decrease of the swollen phase and the increase of the collapsed one happen simultaneously on increasing pressure, it can be interpreted as a transfer from one phase to the other. This interpretation is in line with what is expected from a typical effect of pressure increase that would favour states that occupy the least volume.

Interestingly, the collapsed phases of C10 mix + 2% eicosane and squalane disappear completely at the higher temperatures (T ≥ 20 °C, as shown in the main text and Supplementary Figure 4). We interpret these phases as related to a fraction of flocculated sample, in view of what was found in our previous work[1].

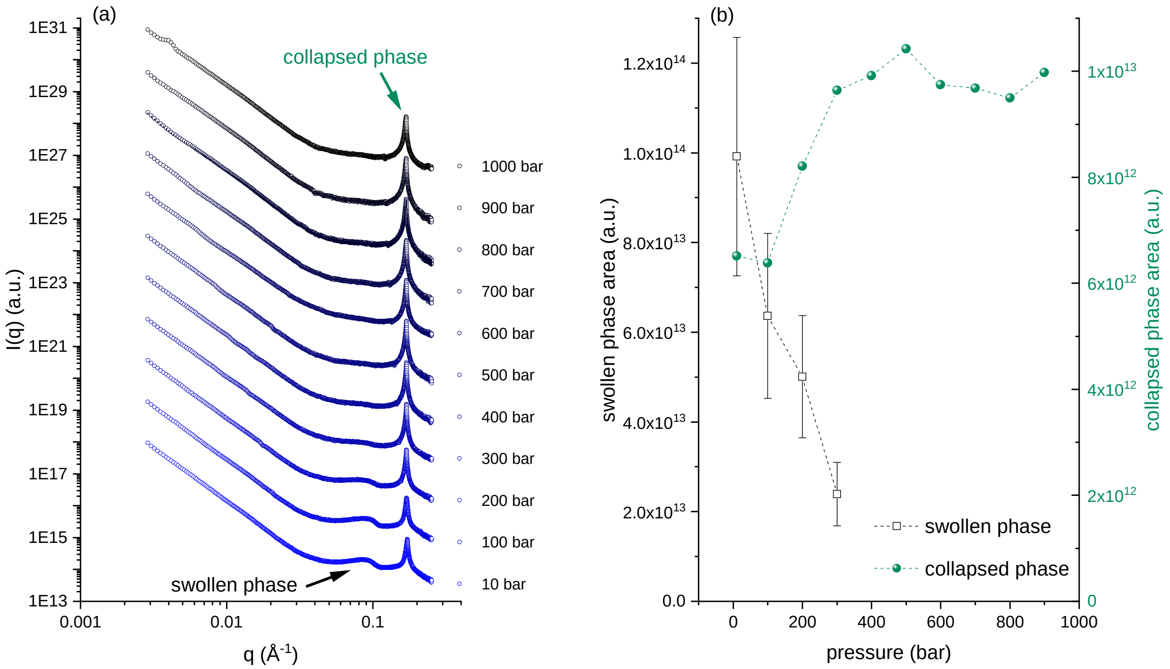

Supplementary Figure 3. C10 mix + 2% squalane at 5 °C. a) SAXS data of sample C10 mix + 2% squalane, T = 5 °C, at all measured pressure points. b) Trend with pressure of the peak integrated intensity corresponding to the swollen and the collapsed phase respectively.

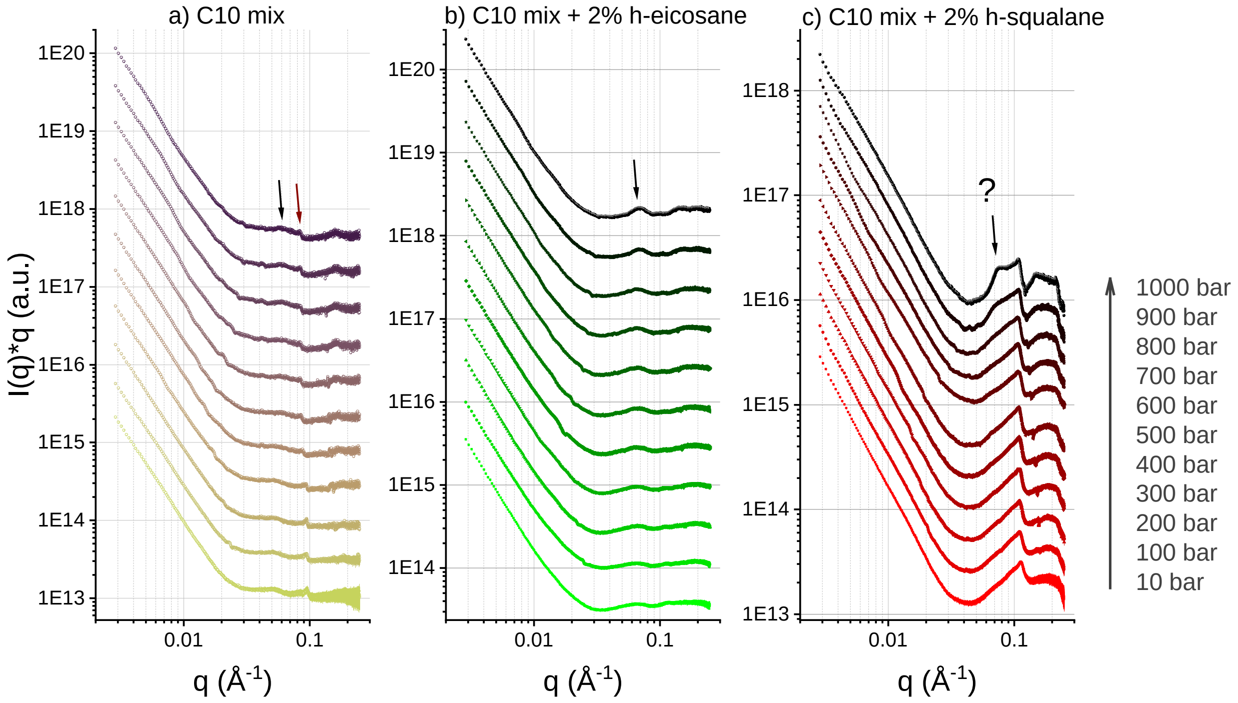

Supplementary Figure 4. SAXS curves obtained for the 3 samples at T = 20°C, in the I*q representation. a) C10 mix; b) C10 mix + 2% h-eicosane; c) C10 mix + 2% h-squalane. Curves have been shifted vertically for clarity. The black arrows point to the 1st order of correlation of the MLVs. Brown arrow indicates the small additional peak on the C10 mix assigned to a different arrangement of the MLV phase. The data of panel (c) were not used in the analysis (as specified in the main text), because a single MLV phase could not be identified (correlation highlighted with an arrow and a question mark).

Supplementary Note 2: SAXS data fitting for T ≥ 20 °C

Supplementary Figure 5 shows the obtained values of the correlation FWHM of the swollen phase investigated in the main text, as function of pressure and at the different temperatures (the peak centers, translated in d-spacing values, are the ones shown in Figure 5 of the main text). The C10 mix (Supplementary Figure 5 (a)) shows a significant increase of the FWHM at T = 20 °C at the intermediate pressure points, which can be an additional sign of phase coexistence in that range. Notably, the C10 mix + squalane sample (Supplementary Figure 5 (c)) shows a counterintuitive FWHM trend between the two measured temperatures: higher temperature should produce more disordered membrane arrangement and therefore lead to a higher correlation FWHM. A reason for this is probably that there is still phase coexistence at T = 35 °C, what is easily visible at the lower temperature T = 20 °C (Figure 4 in the main text). Instead, at T = 35 °C the correlations are probably too close to be resolved, yet slightly shifted leading to an observed higher FWHM.

Supplementary Table 1 lists all fitting parameters for the sample C10 mix + 2% h-eicosane at T = 20 °C (some of the fitting curves are the ones shown in Figure 2 of the main text), as an example of the fits performed. The slope of the power law decay (k) and the peak area (A), were fitted as shared parameters after having verified that the corresponding values were constant upon pressure increasing.

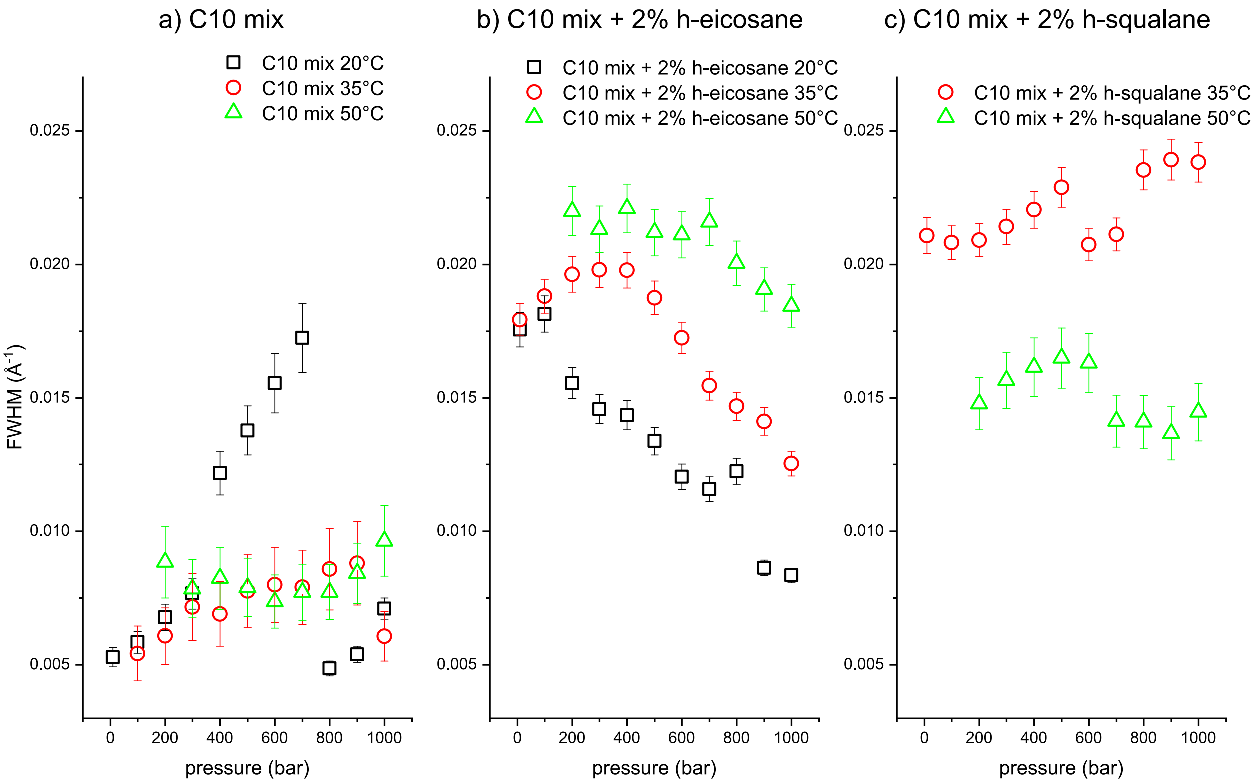

Supplementary Figure 5. MLV peak FWHM of the three measured samples at all the T-p points where the correlation was fitted. a) C10 mix; b) C10 mix + 2% h-eicosane; c) C10 mix + 2% h-squalane.

Supplementary Table 1. Fitting parameters of the SAXS curves for the sample C10 mix + eicosane T = 20 °C.
* shared parameters.

Equation	$m\, q^{-k} + A\, e^{\frac{(q-q_c)^2}{2w^2}}$		
Reduced χ²*	0.1592		
k*	0.935 ± 0.004		
A*	(83.3 ± 1.3) × 10^12		
**pressure (bar)**	**q_c (Å^{-1})**	**w (Å^{-1})**	**m (× 10^{13})**
1	0.0608 ± 0.0004	0.0176 ± 0.0006	3.77 ± 0.05
100	0.0578 ± 0.0005	0.0181 ± 0.0006	3.91 ± 0.05
200	0.0618 ± 0.0004	0.0156 ±0.0006	3.62 ± 0.05
300	0.0618 ± 0.0003	0.0146 ± 0.0006	3.65 ± 0.05
400	0.0619 ± 0.0003	0.0143 ± 0.0005	3.56 ± 0.05
500	0.0624 ± 0.0003	0.0134 ±0.0005	3.47 ± 0.05
600	0.0629 ± 0.0003	0.0121 ± 0.0005	3.58 ±0.05
700	0.0630 ± 0.0003	0.0116 ±0.0005	3.59 ± 0.05
800	0.0629 ± 0.0003	0.0123 ± 0.0005	3.50 ± 0.05
900	0.0675 ± 0.0002	0.0086 ± 0.0003	3.50 ± 0.05
1000	0.0684 ± 0.0002	0.0084 ± 0.0003	3.55 ± 0.05

Supplementary Note 3: FTIR measurements at ambient temperature

Supplementary Figure 6 shows FTIR measurements acquired for a C10 mix sample at 80 mM concentration and extruded with a 100 nm polycarbonate membrane (Avanti Polar Lipid, Alabaster, AL) to give unilamellar vesicles (ULV), as function of hydrostatic pressure. The pressure was controlled by adding small amounts of BaSO4 as pressure probe[2]. Observing the changes in the position of the chain CH_2 symmetric stretching frequency as function of pressure we observe a phase transition starting at 200 > p > 400 bar. Although the high pressure uncertainty does not allow to obtain a precise value of the phase transition temperature, the results are in agreement with what is found on C10 mix at T = 20 °C with SAXS (Figure 5 in the main text). The values of ν_{symm} we obtained (Supplementary Figure 6 (b)) are in perfect agreement with the gel-fluid phase transition observed for the same system at ambient pressure and T ≃ 10 °C by Kapoor et al.[3].

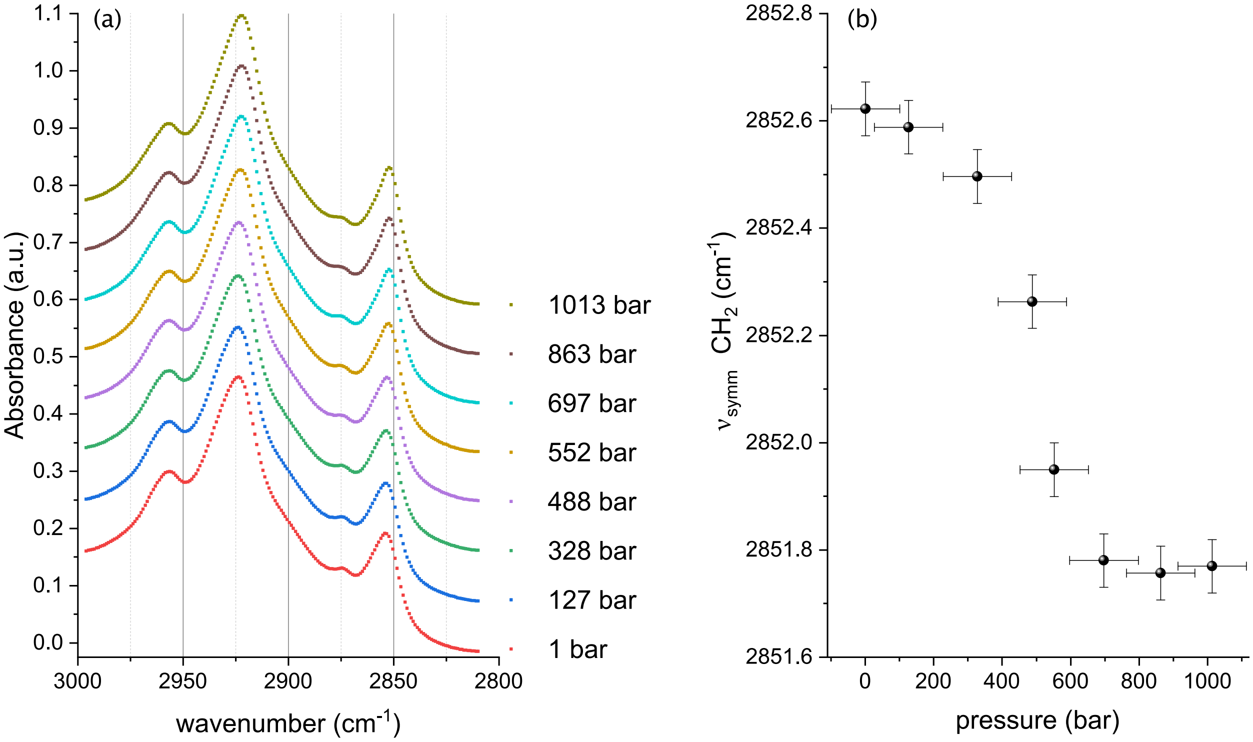

Supplementary Figure 6. Pressure-induced fluid-gel transition of C10 mix membrane at ambient temperature. a) FTIR spectra acquired for C10 mix 80 mM at T = 20 °C and 1 > p > 1013 bar, showing the peaks corresponding to the CH_2 and CH_3 symmetric and asymmetric stretching frequencies. b) Plot of the CH_2 symmetric stretching frequency as function of pressure.

Supplementary References

1. Misuraca, L. *et al.* High-Temperature Behavior of Early Life Membrane Models. *Langmuir* **36**, 13516–13526 (2020).

2. Wong, P. T. T. & Moffat, D. J. A new internal pressure calibrant for high-pressure infrared spectroscopy of aqueous systems. *Appl. Spectrosc.* **43**, 1279–1281 (1989).

3. Kapoor, S. *et al.* Prebiotic cell membranes that survive extreme environmental pressure conditions. *Angew. Chemie* **126**, 8537–8541 (2014).
